# The Critical Need for a Population Health Approach: Addressing the Nation’s Behavioral Health During the COVID-19 Pandemic and Beyond

**DOI:** 10.5888/pcd17.200261

**Published:** 2020-08-06

**Authors:** Arthur C. Evans, Lynn F. Bufka

**Affiliations:** 1 American Psychological Association, Washington, District of Columbia

## Abstract

The COVID-19 global pandemic highlights the necessity for a population health approach to identify and implement strategies across systems to improve behavioral health. Adopting a population health approach helps to address the needs of the total population, including at-risk subgroups, through multiple levels of intervention and to promote the public’s behavioral health and psychological well-being.

**Summary**

**What is known about this topic?**

Behavioral health needs in the United States are not being met by the current health care system, and the COVID-19 pandemic will likely dramatically increase the need for psychological services.

**What is added by this report?**

Adopting a population health approach provides opportunities to target interventions to those populations and communities most in need of psychological health care services, with the potential of preventing development of disorders.

**What are the implications for public health practice?**

Implementing and evaluating population health strategies to promote overall well-being requires system change that translates to policy decisions and programs to meet the needs of local communities.

## Introduction

Calls to bring a population health framework to the nation’s health care system have been increasing. Although this approach had been steadily gaining traction for physical health (1), using this approach with respect to behavioral health (ie, mental health and substance use conditions) has only recently been considered (2,3). However, the need for this approach has never been so apparent as it is during the coronavirus disease 2019 (COVID-19) pandemic. Individuals and communities are grappling with the spread of the virus, the struggle to effectively treat all infected individuals, and the challenges of physical distancing and quarantine, all while attempting to reopen the economy. These challenges, along with the economic impact of prolonged school and business closures and high levels of stress and uncertainty, exact a tremendous psychological toll on many people in the United States (4). The existing capacity of the US health care system to address the resulting behavioral health needs is severely limited (5). A population health approach is needed to address the impact of the COVID-19 pandemic and the inadequacies of the nation’s current approach to behavioral health needs, which have been magnified during the pandemic (6).

The current approach to behavioral health care in the United States is primarily a one-on-one approach that focuses on individuals who have a clinical diagnosis (7). This approach drastically limits the number of people for whom the appropriate level of care is available, let alone addressing the needs of those whose level of psychological distress does not reach the diagnostic threshold. As a result, many people with high levels of stress and uncertainty are left without appropriate psychological support and miss the opportunity for prevention and early intervention.

## The Definition and Application of Behavioral Health

Behavioral health encompasses traditional mental health and substance use disorders, as well as overall psychological well-being (8). Behavioral health can be understood as the behaviors that affect physical and mental health, and good behavioral health results in a “state of mind characterized by emotional well-being, good behavioral adjustment, relative freedom from anxiety and disabling symptoms, and a capacity to establish constructive relationships and cope with the ordinary demands and stresses of life” (9). Obtaining and maintaining behavioral health requires flexibility, the ability to understand and manage emotions, engaging in behaviors that are healthy for the body and the mind, awareness of one’s relationship to others and recognition of one’s responses, and effectively employing strategies to deal with the demands of living.

The manifestation of behavioral health varies over the lifespan and across cultures. Similarly, the large number of factors that influence behavioral health must also be acknowledged: genetics, family environment, discrimination, socioeconomic status, traumatic experiences, physical health, loneliness, culture, and a host of others (10). Supporting behavioral health often means addressing social determinants of health through an array of social and community factors (11). For instance, when individuals and communities lack economic stability, physical survival alone can be a challenge. The focus is on getting what is needed to live, which will not necessarily include what is needed to thrive. Integrating behavioral health with community access to job training programs is one example of increasing access to behavioral health services and to psychological skill development to help individuals navigate the challenges of seeking employment.

We need to be as concerned about a population’s psychological well-being as we are about its physical well-being. Psychological well-being is neither a categorical nor a permanent state. That is, people are not either mentally healthy or unhealthy (eg, meeting diagnostic criteria for a psychological disorder, such as depression or schizophrenia; developing a substance use problem). A person’s or population’s overall psychological well-being falls on a continuum and changes over time. To truly recognize and support degrees of mental wellness on that continuum requires changing how we identify and meet the behavioral health needs of the population.

## Specialist Health Care Framework Is Insufficient

How behavioral health is addressed within our health care system must change. Currently, one must typically have a diagnosis to have care covered by insurance; therefore, early intervention and prevention is difficult, and in many places in the United States, access to services is limited (12). Furthermore, specialist behavioral health care professionals, such as psychologists and psychiatrists, work in settings distinct from where most of individuals live, work, play, and worship, creating both physical and psychological barriers to access.

Although more integration of professionals who specialize in behavioral health care into primary care and other settings has occurred, the trend is not universal and it does not go far enough in reaching people in other settings. In instances in which this integration has occurred, the behavioral health expert has the capacity to immediately meet with individuals who have identified behavioral health needs, triage the concerns, and determine appropriate next steps, thereby reducing the number of individuals who are “lost” in the transition to specialty care. Also, the psychologist or other behavioral health care professional frequently provides consultation and support to nonbehavioral health care professionals, helping to educate them as well as reduce the stigma often associated with patients who have behavioral health care needs (13). Integrated care improves on our current approach by providing a range of interventions and reaching people “where they are” (13). This approach, similar to a population health approach, emphasizes addressing behavioral health needs — regardless of whether the person has a diagnosis — and building the capacity of the setting to address behavioral health needs along a continuum.

Addressing behavioral health within the health care system alone is not sufficient. Many individuals do not have a regular primary care provider. Of those who do, the behavioral health needs being addressed are those further along the continuum toward distress, impairment, and disorder. Because only 50% of individuals with behavioral health concerns actually enter any form of treatment (14), we must develop new strategies to reach people wherever they are — at work, in school, and in the community. Furthermore, we must engage the communities themselves, which have the wisdom to address many of these problems but may need the resources and expertise of mental health professionals to do so.

## Scope of Needs During the COVID-19 Pandemic

Behavioral health needs have long been insufficiently met in the United States, and the population is now facing increasing psychological stress and significant growing needs as the pandemic unfolds (15). According to a survey conducted by the American Psychological Association (APA), the average stress level reported by US adults in May 2020 was significantly higher than that reported in the 2019 survey (data collected in August), and it is the first significant increase in average reported stress since APA first started surveying American households about stress more than a decade ago (16). Furthermore, some groups in the APA survey, such as parents with children younger than 18 and Hispanic adults, reported even higher levels of stress. Stress that is not addressed can become chronic and result in physical and behavioral health problems such as cardiovascular disease, obesity, inflammation, and depression (17).

Analyses from previous pandemics (18,19), as well as studies about COVID-19 coming from China (20) and Italy (21), indicate that we should expect an increase in a variety of behavioral health symptoms, especially among front-line health care workers. Emerging data suggest that health care workers treating individuals with COVID-19 are reporting significant distress and symptoms of depression, anxiety, and insomnia (22). At a minimum, those on the front lines of addressing COVID-19 need onsite emotional support and the capacity to meet their own basic needs such as obtaining food, transportation, and personal protective equipment. Some of those on the front lines experiencing distress will want and benefit from more focused, brief psychological interventions intended to provide them with skills that enable them to cope with highly stressful work situations (eg, Psychological First Aid, Skills for Psychological Recovery) (23). Unfortunately, many hospitals are not set up to provide this kind of psychological support (24,25).

Furthermore, a 2020 systematic review of the psychological impact of quarantine indicated that individuals experience an array of negative effects, including anger, confusion, and posttraumatic stress symptoms (26). These effects are heightened when quarantine is of a longer duration, people have fears of infection, receive inadequate or unclear information, and face financial loss. If the pandemic is similar to other community traumas (27), most individuals will adapt and demonstrate resilience, but a minority will develop a behavioral health condition that requires intervention.

The long-term population health needs resulting from the pandemic could be substantial. Although humans are remarkably resilient, some individuals benefit from psychological intervention. In addition to workers on the front lines (eg, health care professionals, essential workers) who may develop disorders such as depression or posttraumatic stress disorder as a result of their experiences treating individuals with COVID-19, many other segments of the US population (and worldwide) are also likely to need interventions in some form. In the current environment of quarantine and physical distancing, patients with COVID-19 are typically separated from their families and do not have the benefit of the close emotional support and physical help of their loved ones.

The families and friends of patients with COVID-19 experience high levels of stress, which is magnified in cases in which they are unable to be present when their loved ones die. Furthermore, because traditional funerals and other rituals are not possible in the current environment, survivors must create new ways to mourn. Individuals who survive COVID-19 may have major behavioral health needs that we are only beginning to understand. For instance, research makes clear that the experience of being on a ventilator and staying in an intensive care unit for an extended period of time can be traumatic (28,29). Some individuals may face cognitive challenges as they recover from the infection, which necessitates specialized behavioral health care (30).

In addition to the large numbers of individuals who have had direct experience with COVID-19, the US population has also experienced some degree of stress as a result of the nation’s sweeping efforts to reduce transmission of the virus. Many individuals have struggled to cope with the uncertainty of stay-at-home orders, changes in work and financial status, facilitating their children’s online schooling, virus-related discrimination, and major disruptions in routines and plans. Each of these factors poses the potential for the development of ongoing stress and its fallout. Of particular concern are people facing both significant financial distress and experiencing discrimination, as both of these stressors are linked to the development of future behavioral health problems (31,32).

## Adopting a Population Health Framework

In the face of this kind of population distress, the importance of using public health strategies, rather than relegating behavioral health to treatment by specialist providers only, cannot be overstated. Promoting population behavioral health has the potential to increase overall resiliency and reduce the number of individuals who ultimately develop behavioral health problems, and improvements in behavioral health can also lead to improvements in physical health (33). This crisis, although difficult, can provide an opportunity to make this shift. Philadelphia (34) and New York City (35) have adopted a population health approach to behavioral health and provide models for how to begin. Key aspects of this work include the necessity of reimagining what a behavioral health system is and how one operates and to establish a broad, evidence-based vision of what that entails.

This change needs to happen both at the national and the local level. National leadership can highlight issues, advocate for resources, and encourage solutions, but implementation must take place at the local level to best meet community needs. Unfortunately, many local health governments are not actively engaged in systematic activities to promote behavioral health. Although local leaders often recognize the priority of doing so, they often do not control the behavioral health resources in their communities, which are often administered at the state or county level. Consequently, local leaders cite as barriers limited resources, knowledge, and data along with the challenges of communicating and collaborating with local behavioral health agencies (36). Increasing partnerships between these local governments and behavioral health funding agencies is essential for success.

The American Psychological Association (APA) is using a population health framework to tackle the emerging behavioral health issues associated with this pandemic. APA has identified several principles to guide this work (Box), conceptualized as taking place across 3 broad levels of the population: 1) those with behavioral health conditions requiring clinical intervention, 2) those who are experiencing subclinical psychological distress or who are at great risk for experiencing clinically significant behavioral health problems, and 3) those who are relatively healthy.

**Box. Principles Guiding Population Health Framework for Behavioral Health at the American Psychological Association**

• Use data and the best available science to inform policies, programs, and resources.

• Prevent when possible and otherwise intervene at the earliest moment.

• Strategize, analyze, and intervene at the community/population level (in addition to the individual).

• Reach broad and diverse audiences through partnerships and alliances.

• Utilize a developmental approach (eg, change over time, age-appropriate interventions).

• Consider the “whole person” and the structural/systemic factors impacting individual behavior.

• Be culturally sensitive while also thinking transculturally.

• Recognize that inherent in every community is the wisdom to solve its own problems.

• Champion equity by addressing systemic issues (eg, social determinants of health, access to treatment).

Strategies and interventions must be tailored to achieve the health goals at each of these levels. *Indicated* approaches to behavioral health target the first level. These approaches are often provided by specialists, such as psychologists, to individuals with clear problems or disorders and use evidence-based strategies to reduce symptoms and improve functioning. *Selective* approaches to behavioral health are designed to reduce risk or mitigate the impact of risk factors that lead to psychological distress, for example using targeted, scalable interventions designed to build people’s ability to adapt and cope. *Universal* approaches are intended to promote general behavioral wellness, with a focus on messages to the public to destigmatize mental illness, promote psychoeducation about responses to stress, and focus attention on the foundation necessary to support and maintain psychological well-being. 

A population health approach has, as its goal, optimal behavioral health and wellness across the continuum of need. This approach addresses the need to “get upstream” as it promotes intervention before individuals need clinical services. It also shifts the goal of practitioners to behavioral wellness and not just the absence of psychopathology. Because this is a significant paradigmatic shift for most behavioral health professionals and the systems in which they work, we will need to develop leaders and professionals who can work from this public health perspective. From a systems perspective, individual localities should determine their own needs and collaboratively work with local experts — members of the public, scientists, providers, policy makers, and others — to design and implement the programs that each community needs.

## Implications for Public Health

The pandemic has elevated stress levels nationwide, with serious implications. Chronic stress is linked to greater risk for a range of adverse health outcomes, so adopting a rigorous, evidence-based approach to identifying needs and designing interventions is critical. In the United States, there have been some effective public education campaigns to encourage handwashing, physical distancing, and mask wearing to slow the spread of the coronavirus. Similarly, key messages can be developed and used to increase the public’s capacity to handle stress, cope with the current uncertainty, and manage distress to slow the development of behavioral health problems. The opportunity to act is now, before a behavioral health pandemic develops and accelerates and too many lives are disrupted or lost.

Using a population approach to behavioral health holds much promise. It will allow us to address many long-standing issues that affect our current behavioral health system by placing a greater emphasis on prevention and early intervention and by reaching underserved subgroups. It will also enable us to simultaneously and effectively address the potential surge in need caused by the COVID-19 pandemic. The challenge will be reorienting and training the workforce to adopt this perspective, develop new interventions, and build the service infrastructure to meet a broader range of behavioral health needs. Furthermore, we need to develop a fiscal and regulatory policy framework to support this work. Finally, evaluation of these changes can be essential to determine how future population health approaches can be effective at improving not only the psychological well-being of those impacted by COVID-19 but also the overall behavioral health of the US population. Although there are important examples of the successful implementation of a population mental health approach, these are rare exceptions. The behavioral health pandemic that is likely to emerge as a result of COVID-19 creates urgency and should spur immediate action. We have a window of opportunity where the public and policy makers can see firsthand that behavioral health concerns are affecting a large proportion of the population and that we need an approach and the resources to address the full range of these concerns. Action must be taken for the health and well-being of our nation.
